# Supplementary material for: Acetate-Mediated Odorant Receptor OR51E2 Activation Results in Calcitonin Secretion in Parafollicular C-Cells: A Novel Diagnostic Target of Human Medullary Thyroid Cancer
Source: Biomedicines. 2023 Jun 11;11(6):1688. doi: 10.3390/biomedicines11061688 (PMC10296248; doi:10.3390/biomedicines11061688)
Supplement: Supplementary file 1 [file biomedicines-11-01688-s001.zip › biomedicines-2322517-supplementary.pdf]

Supplementary Material

1 Supplementary Figure and Tables

1.1 Supplementary Figure

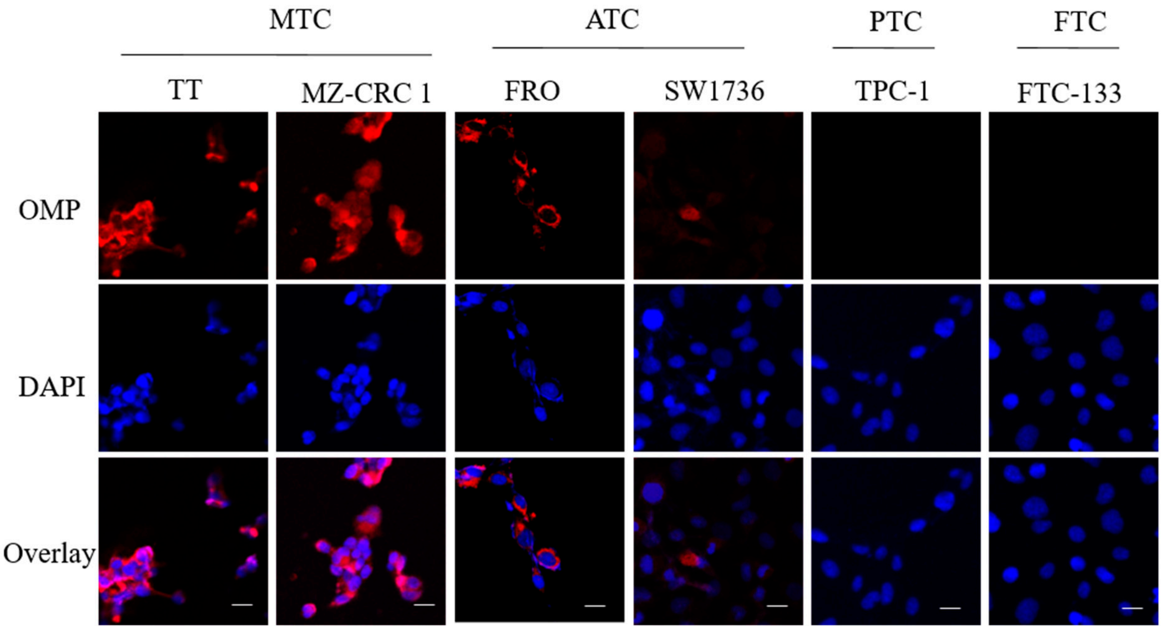

Supplementary Figure S1. Expression of olfactory marker protein in multiple thyroid cancer cell lines. Immunofluorescence staining of medullary thyroid cancer (MTC) cell lines (TT and MZ-CRC-1), ATC cell lines (FRO and SW1736), a PTC cell line (TPC-1), and an FTC cell line (FTC-133) labeled with an olfactory marker protein-specific antibody (red). Nuclei are stained with DAPI (blue). All pictures are shown at 20× magnification.

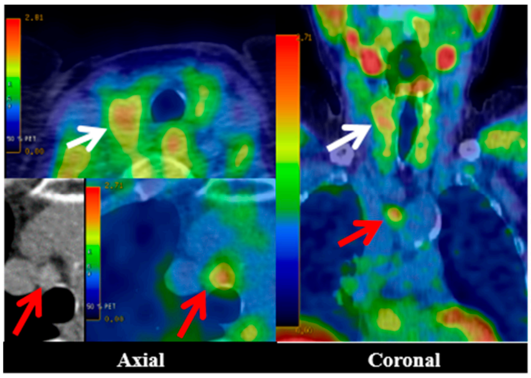

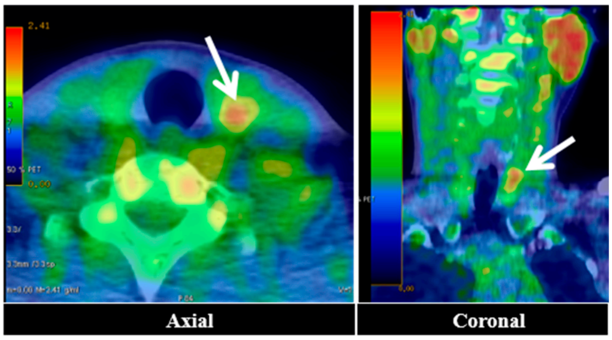

Supplementary Figure S2. C^{11}-acetate-OR51E2 is a diagnostic target for patients with MTC. C^{11}-acetate positron electron tomography/computed tomography of a 31-year-old male patient with MTC presenting with mediastinal lymph node (LN) metastasis. C^{11}-acetate uptake is observed in the primary tumor (white arrow) and mediastinal LN metastasis (red arrow).

1.2 Supplementary Tables

Gene ID	Expression values (RPKM)	Exon length	Exons	Chromosome	Chromosome region start	Chromosome region end
OR2W3	6.182893106	945	1	NC_000001	248058889	248059833
OR2T33	2.226316839	963	1	NC_000001	248436154	248437116
OR2T8	1.982135615	939	1	NC_000001	248084320	248085258
OR4N4	1.719182291	1343	1	NC_000015	22382473	22383815
OR6F1	1.372416376	927	1	NC_000001	247875131	247876057
OR56A3	1.018937123	948	1	NC_000011	5968577	5969524
OR10H5	0.994084998	948	1	NC_000019	15904859	15905806
OR13A1	0.907018843	2078	4	NC_000010	45798102	45811056
OR1C1	0.822723679	945	1	NC_000001	247920764	247921708
OR51E2	0.651384457	2785	2	NC_000011	4701401	4719076
OR52E6	0.60025005	942	1	NC_000011	5862186	5863127
OR10AD1	0.518612268	954	1	NC_000012	48596122	48597075
OR13G1	0.407962155	924	1	NC_000001	247835420	247836343
OR2AG2	0.322058452	951	1	NC_000011	6789238	6790188
OR10H2	0.274750023	1029	1	NC_000019	15838834	15839862
OR56B4	0.269956207	960	1	NC_000011	6129009	6129968
OR1E1	0.224379185	945	1	NC_000017	3300760	3301704

OR2C3	0.22339722	2742	2	NC_000001	247693434	247697141
OR1L8	0.202665071	930	1	NC_000009	125329827	125330756
OR14I1	0.201365936	936	1	NC_000001	248844670	248845605
OR52B6	0.186982654	1008	1	NC_000011	5602107	5603114
OR52E4	0.17563227	939	1	NC_000011	5905523	5906461
OR56A4	0.171656207	1098	1	NC_000011	6023281	6024378
OR3A3	0.170723293	966	1	NC_000017	3323862	3324827
OR1J1	0.170194738	969	1	NC_000009	125239237	125240205

Supplementary Table S1. RNA Sequence data for known OR gene expression in medullary thyroid cancer cell line (MZ-CRC-1) (The RPKM values are listed in descending order)

Gene ID	Expression values (RPKM)	Exon length	Exons	Chromosome	Chromosome region start	Chromosome region end
OR2A1	0.395989851	933	1	NC_000007	144015218	144016150
OR56B4	0.329873688	960	1	NC_000011	6129009	6129968
OR51B4	0.188566596	933	1	NC_000011	5322244	5323176
OR2A7	0.188566596	933	1	NC_000007	143955789	143956721
OR52E6	0.149412003	942	1	NC_000011	5862186	5863127
OR1J2	0.112059002	942	1	NC_000009	125273081	125274022
OR10AD1	0.110649455	954	1	NC_000012	48596122	48597075
OR2A42	0.075426638	933	1	NC_000007	143929004	143929936
OR1J4	0.074706002	942	1	NC_000009	125281420	125282361
OR1Q1	0.07446884	945	1	NC_000009	125377017	125377961
OR1E1	0.07446884	945	1	NC_000017	3300760	3301704
OR1K1	0.073999005	951	1	NC_000009	125562402	125563352
OR7C1	0.073076899	963	1	NC_000019	14909986	14910948
OR52W1	0.073076899	963	1	NC_000011	6220454	6221416
OR52H1	0.073076899	963	1	NC_000011	5565791	5566753

OR3A3	0.072849952	966	1	NC_000017	3323862	3324827
OR1J1	0.07262441	969	1	NC_000009	125239237	125240205
OR52B6	0.069814537	1008	1	NC_000011	5602107	5603114
OR52N5	0.068059046	1034	1	NC_000011	5798864	5799897
OR51E1	0.056642831	3106	2	NC_000011	4665156	4676718
OR2B6	0.056029501	942	1	NC_000006	27925019	27925960
OR56A3	0.055674884	948	1	NC_000011	5968577	5969524
OR1E2	0.054300196	972	1	NC_000017	3336164	3337135
OR51B5	0.051367192	1370	4	NC_000011	5362113	5368140
OR4K13	0.038455221	915	1	NC_000014	20502003	20502917

Supplementary Table S2. RNA Sequence data for known OR gene expression in medullary thyroid cancer cell line (TT) (The RPKM values are listed in descending order)
